# Survey on academic self-efficacy, academic stress, critical thinking, performance expectations, and students’ AI dependency

**DOI:** 10.1016/j.dib.2026.112952

**Published:** 2026-06-09

**Authors:** Quynh Pham Viet, Duy Van Nguyen, Nguyen Thi Xiem, Dat Ngoc Nguyen

**Affiliations:** a Faculty of Education, Hanoi Metropolitan University, Hanoi, Viet Nam; b Faculty of Business Administration, Phenikaa University, Duong Noi, Hanoi 12116, Viet Nam; c Foreign Trade University, Hanoi, Viet Nam

**Keywords:** Academic self-efficacy, Academic stress, Critical thinking, Performance expectations, Students, AI dependency

## Abstract

In the context of AI development, not only in business and society but also in education, students are increasingly using AI in their studies. This could become a concern due to the decline in critical thinking and the increased dependence on AI. This study focuses on academic self-Efficacy, academic stress, critical thinking, performance expectations, and Students’ AI dependency. Data were collected from 1580 university students in Vietnam. The study examined the reliability and discriminant validity of the constructs using Confirmatory Factor Analysis **(**CFA) in SmartPLS. The results showed that all constructs met the requirements for reliability and discriminability. The data will contribute not only to academic research by helping researchers conduct AI-related studies, but also to policymakers who can assess the current state of academic pressure and whether the use of AI in learning reduces critical thinking, learning performance expectations, and dependence on AI.

Specifications Table

**Table:** 

Subject	Social Sciences (General)
Specific subject area	Education
Type of data	Table, Raw, analysed
Data collection	The data were collected from December 2025 to February 2026 by an online system.
Data source location	Region: Asia Country: Vietnam
Data accessibility	The dataset is provided as a supplementary file. Direct URT to data: https://data.mendeley.com/datasets/2rrz65cxp9/1 Data identification number DOI: 10.17632/2rrz65cxp9.1

## 1 Value of the Data

• Universities can use this dataset and apply multivariate analytical techniques such as PLS-SEM to identify the factors that contribute to increased AI dependence among students.
• Lecturers can use this dataset to analyze and identify effective strategies for redesigning teaching methods and course assessment practices in order to enhance students' self-directed learning abilities and reduce their dependence on AI.
• The data will provide universities with implications related to developing students’ critical thinking and reducing their dependence on AI.
• The data may serve as a practical dataset for courses related to data analysis, such as SEM modeling and analyses of differences in students’ dependence on AI by gender, major, type of university, or experience in using AI.
• The data will help researchers compare the findings with previous studies conducted in different countries in order to identify differences among educational systems.

## 2 Background

In the context of internet development, opportunities for growth have been created for businesses and other sectors [1]. AI is significantly affecting business sectors and social life, and education has been particularly influenced, as students have increasingly learned to use AI for studying and completing assignments. The development of AI has made it necessary for teaching approaches and students’ learning methods to change in order to adapt [2]. The advancement of generative AI has also expanded the application of AI in education through tools that can generate content such as text, images, and code based on complex user prompts [3]. Therefore, AI can be considered an important driver of educational innovation in the current context of digital transformation [3,4]. Many studies have highlighted the various benefits AI offers students in learning. AI helps students access information more quickly and improves their learning efficiency [5,6]. In addition, AI can support the development of creative thinking skills, problem-solving abilities, and effective self-directed learning among learners [[7], [8], [9]]. However, the use of AI in learning also poses many challenges, particularly the risk of technological dependence and the decline of students’ independent thinking abilities [10,11].

When students have access to and use AI for learning, it can explain concepts and quickly search for materials. This helps students save time in finding relevant resources [9]. Even so, the risk of dependence on AI has become more common when students use it passively. Instead of exploring and synthesizing materials on their own, students may easily accept and copy content generated by AI to complete their assignments. This may weaken critical thinking, problem-solving, and learning autonomy, which are essential competencies that need to be cultivated in students [12,13]. Therefore, AI should be regarded as a learning support tool rather than a substitute for students’ independent thinking process. Accordingly, research on AI dependence plays an important role for universities in adjusting teaching and assessment methods to enhance students’ learning effectiveness and critical thinking.

Learning self-efficacy refers to an individual’s belief in their ability to complete academic tasks and achieve strong learning outcomes [14]. According to social cognitive theory, beliefs about one’s own capabilities influence behavior, motivation, and academic performance [4,15]. From this perspective, high self-efficacy for learning may help students enhance their critical thinking in both academic settings and social life. Students with higher self-efficacy are more likely to question information they receive. This is especially important when using AI in learning, as information generated by AI should always be verified for accuracy and source reliability [7].

Critical thinking is a purposeful thinking process used to evaluate information and make decisions about problems to be solved [7]. In the context of the widespread use of AI in education, some studies have also shown that excessive reliance on AI may reduce learners’ ability to analyze and evaluate information [13,16]. Therefore, data showing the relationships among critical thinking, learning effectiveness, and the level of AI dependence has become an important issue in the current higher education context [17]. The data will be provided to universities to support improvements in learning effectiveness, critical thinking, and reducing AI dependence. In addition, researchers may use the data as a reference for their own studies on students’ dependence on AI.

## 3 Data Description

After being designed, the questionnaire was distributed online to students studying at universities in Vietnam. A convenience sampling method and probabilities based on the balanced male-female ratio were used in this study. Students were informed in advance about the research content and agreed to participate in the survey voluntarily. Specifically, the research objectives and scope were clearly explained, and students who found the study appropriate and had no privacy concerns could choose whether or not to participate. If a student chose not to participate, a thank-you message was displayed, and the survey ended. Students who voluntarily agreed to participate were then directed to the subsequent survey questions. This study was initiated under Degree No. 022,026/QDNC-QAglobal issued by the Quantitative Analysis Center, QAglobal, Vietnam. The survey, conducted from December 2025 to February 2026 via an online Google Form, collected 1580 responses for analysis (the response rate for online surveys reached 100%, and participants were required to answer all questions before submitting responses). The number of responses was considered adequate for analysis according to the sampling guidelines of Hair et al [18] and Comrey and Lee [19]. Information about the survey participants is presented in Table 1.

**Table 1:** Respondents’ profiles (*n* = 1580).

		n(%)				n(%)
Gender				Major		
	Female	756(47.8%)			Education	726(45.9%)
	Male	824(52.2%)			Engineering	269(17.1%)
Year					Economic	511(32.3%)
	1	184(11.7%)			Language	14(0.9%)
	2	791(50%)			Others	60(3.8%)
	3	538(34.1%)		Time to use AI		
	4	40(2.5%)			Under 1 year	264(16.7%)
	5	27(1.7%)			1 to Under 2 years	805(50.9%)
Type of university					For 2 years	511(32.4%)
	Private university	60(3.8%)				
	Public university	1520(96.2%)				

*n* = 1580						

## 4 Experimental Design, Materials and Methods

The measurement scales were developed based on the studies of Zhang et al [4] and Acosta-Enriquez [5]. Specifically, academic self-efficacy was measured using 6 items adapted from Zhang et al [4]. Performance expectations were measured with 5 items, also adopted from Zhang et al [4]. Critical thinking was measured using 4 items from the study by Acosta-Enriquez [5]. Academic stress was measured with 4 items adapted from Zhang et al [4]. AI dependency was developed with reference to Acosta-Enriquez [5] and consisted of 5 items. The details are presented in Table 1. The questionnaire was translated from English into Vietnamese and adjusted to fit the Vietnamese context. After that, it was back-translated from Vietnamese into English to examine whether any changes in meaning had occurred. The back-translation process was used to minimize potential changes in content during translation from English to Vietnamese. A five-point Likert scale, ranging from 1 = strongly disagree to 5 = strongly agree, was used in this study. The questionnaire, once designed, was officially distributed to students in Vietnam. Students were provided with full information before answering and agreed to answer voluntarily. This study was commissioned by the Quantitative Analysis Center of QAglobal, Vietnam, under Degree No. 022,026/QDNC-QAglobal.

After being cleaned and coded in Excel, the data were imported into SmartPLS for analysis. In the CFA and PLS-SEM analyses employed in the subsequent stages of the study, latent constructs were not created by summing or averaging item scores. Instead, latent variable scores were generated using the PLS-SEM weighting algorithm, which estimates construct scores as weighted combinations of standardized indicators, as presented in Eq. (1):

(1) $$Z_{i}=\frac{X_{i}-\bar{X}}{SD_{i}}$$

After the items were standardized, the weights $w_{i}$ were applied to construct the latent variable scores, as presented in Equation (2):

$$Latent\;variable=\sum_{i=1}^{k}w_{i}Z_{i}$$

First, the study used confirmatory factor analysis **(**CFA) to assess reliability, in which Cronbach’s alpha for each construct must be greater than 0.6, composite reliability (CR) must be greater than 0.7, and average variance extracted (AVE) must be greater than 0.5. In addition, the constructs must demonstrate convergent validity, with outer loadings exceeding 0.7 [18]. Next, discriminant validity was examined using two criteria. First, the square root of AVE should be greater than the corresponding correlation coefficients. Second, an HTMT value below 0.85 is considered evidence of adequate discriminant validity [20].

The CFA results indicate that all constructs achieved acceptable reliability, with Cronbach’s alphas ranging from 0.863 to 0.918. Composite reliability (CR) values were also above 0.7, ranging from 0.907 to 0.938, while average variance extracted (AVE) values exceeded 0.5, ranging from 0.671 to 0.752. In addition, all outer loading values were above 0.7, indicating that all constructs satisfied the requirement for convergent validity (Tables 2 and 3).

**Table 2:** Reliability and convergent validity.

Code	Contents	Outer loading
**Academic self-efficacy adapted from Zhang et al [**4**]; α = 0.902; CR = 0.924; AVE = 0.671**		
**ASE1**	You are confident in your ability to understand even the most complex concepts presented in class.	0.807
**ASE2**	You are confident that you can do very well on assignments and exams.	0.832
**ASE3**	You can master the skills taught in your courses.	0.819
**ASE4**	You are confident in your ability to learn independently and grasp the subject matter.	0.834
**ASE5**	You are capable of achieving good academic results through your own abilities.	0.812
**ASE6**	You can achieve your academic goals even when faced with challenges.	0.811
**Academic stress adapted from Zhang et al [**4**]; α = 0.871; CR = 0.912; AVE = 0.721**		
**AST1**	You are worried about finding a good job after graduation.	0.82
**AST2**	You feel overwhelmed by the amount of academic work you have to handle.	0.872
**AST3**	You feel anxious facing deadlines and academic demands.	0.857
**AST4**	You worry that you won't be able to meet academic expectations.	0.847
**Critical thinking adapted from Acosta-Enriquez [**5**]; α = 0.863; CR = 0.907; AVE = 0.71**		
**CT1**	You carefully analyze information before accepting it as true.	0.841
**CT2**	You evaluate different viewpoints before reaching a conclusion.	0.862
**CT3**	You can determine the relevance and validity of arguments.	0.856
**CT4**	You develop creative solutions to complex problems.	0.809
**Performance expectations adapted from Zhang et al [**4**]; α = 0.913; CR = 0.935; AVE = 0.74**		
**PE1**	Using AI has improved your academic performance.	0.838
**PE2**	AI helps you complete learning tasks faster.	0.871
**PE3**	AI tools increase your learning productivity.	0.877
**PE4**	Using AI makes your learning tasks more efficient.	0.879
**PE5**	AI tools help you achieve better grades.	0.839
**AI dependency adapted from Acosta-Enriquez [**5**]; α = 0.918; CR = 0.938; AVE = 0.752**		
**AID1**	Are you worried about not being able to use AI for your academic tasks?	0.853
**AID2**	Do you find it difficult to control the amount of time you spend using AI?	0.871
**AID3**	Are you overly reliant on AI to complete your academic assignments?	0.886
**AID4**	Do you struggle to complete academic tasks without AI support?	0.874
**AID5**	Do you feel the need to use AI more and more frequently to maintain your academic performance?	0.853

**Table 3:** Discriminant validity.

Fornell & Larcker					
	*AID*	*AS*	*AST*	*CT*	*PE*
AID	**0.867**				
AS	0.390	**0.819**			
AST	0.499	0.437	**0.849**		
CT	0.356	0.720	0.514	**0.842**	
PE	0.512	0.552	0.534	0.651	**0.861**


HTMT					
	*AID*	*AS*	*AST*	*CT*	*PE*
AID					
AS	0.428				
AST	0.553	0.491			
CT	0.397	0.815	0.593		
PE	0.553	0.606	0.60	0.733	

*Notes*: Fornell & Larcker: 1st value = Correlation between variables (2-tailed *t*-test); Square root of AVE (bold diagonal).

The results of the discriminant validity analysis indicate that all constructs exhibit adequate discriminant validity, as the square roots of the AVEs are all greater than the corresponding correlation coefficients [20]. The square roots of AVE ranged from 0.819 to 0.867, while the correlation coefficients ranged from 0.356 to 0.720. In addition, the study also examined HTMT values, all of which were below 0.85, further confirming that the constructs satisfied the requirement for discriminant validity.

## Ethics Statement

Participants were informed about the study’s objectives, target respondents, and survey duration before participating. Participation was voluntary, and no personal identifying information was collected to protect respondents’ privacy. This study was initiated under Degree No. 022,026/QDNC-QAglobal issued by the Quantitative Analysis Center, QAglobal, Vietnam.

## Declaration of AI using assisted technologies

The author(s) used OpenAI’s ChatGPT (version November 2025) to improve the clarity and grammar of the Abstract section. All content was reviewed and verified by the authors.

## CRediT Author Statement

**Quynh Pham Viet:** Conceptualization, Methodology, Writing – original draft, Visualization. **Duy Van Nguyen:** Conceptualization, Methodology, Writing – original draft, Visualization. **Nguyen Thi Xiem:** Data collection, Methodology, Writing – original draft. **Dat Ngoc Nguyen:** Methodology, Writing – original draft.

## Data Availability

Mendeley DataSurvey on Academic Self-Efficacy, Academic Stress, Critical Thinking, Performance Expectations, and Students’ AI Dependency (Original data).

## Appendix Supplementary materials

Image, application 1

Image, application 2
