# Supplementary material for: Survey on academic self-efficacy, academic stress, critical thinking, performance expectations, and students’ AI dependency
Source: Data Brief. 2026 Jun 9;67:112952. doi: 10.1016/j.dib.2026.112952 (PMC13280133; doi:10.1016/j.dib.2026.112952)
Supplement: Supplementary file 1 [file mmc1.docx]

**APPENDIX.**

**QUESTIONNAIRE**

**Question content**

Please circle the corresponding answers to your level of agreement with those statements. In which:

| **1. Strongly disagree** |  | **5. Strongly agree** |
| --- | --- | --- |

| **Code** | **Contents** | **Levels** |
| --- | --- | --- |
| **Academic self-efficacy** | |  |
| ﻿ASE1 | You are confident in your ability to understand even the most complex concepts presented in class. | 1 2 3 4 5 |
| ﻿ASE2 | You are confident that you can do very well on assignments and exams. | 1 2 3 4 5 |
| ﻿ASE3 | You can master the skills taught in your courses. | 1 2 3 4 5 |
| ﻿ASE4 | You are confident in your ability to learn independently and grasp the subject matter. | 1 2 3 4 5 |
| ﻿ASE5 | You are capable of achieving good academic results through your own abilities. | 1 2 3 4 5 |
| ﻿ASE6 | You can achieve your academic goals even when faced with challenges. | 1 2 3 4 5 |
| **Academic stress** | |  |
| AST1 | You are worried about finding a good job after graduation. | 1 2 3 4 5 |
| AST2 | You feel overwhelmed by the amount of academic work you have to handle. | 1 2 3 4 5 |
| AST3 | You feel anxious facing deadlines and academic demands. | 1 2 3 4 5 |
| AST4 | You worry that you won't be able to meet academic expectations. | 1 2 3 4 5 |
| **Critical thinking** | |  |
| CK1 | You carefully analyze information before accepting it as true. | 1 2 3 4 5 |
| CK2 | You evaluate different viewpoints before reaching a conclusion. | 1 2 3 4 5 |
| CK3 | You can determine the relevance and validity of arguments. | 1 2 3 4 5 |
| CK4 | You develop creative solutions to complex problems. | 1 2 3 4 5 |
| **Performance expectations when using AI** | |  |
| PE1 | Using AI has improved your academic performance. | 1 2 3 4 5 |
| PE2 | AI helps you complete learning tasks faster. | 1 2 3 4 5 |
| PE3 | AI tools increase your learning productivity. | 1 2 3 4 5 |
| PE4 | Using AI makes your learning tasks more efficient. | 1 2 3 4 5 |
| PE5 | AI tools help you achieve better grades. | 1 2 3 4 5 |
| **AI dependency** | |  |
| AID1 | Are you worried about not being able to use AI tools for your academic tasks? | 1 2 3 4 5 |
| AID2 | Do you find it difficult to control the amount of time you spend using AI? | 1 2 3 4 5 |
| AID3 | Are you overly reliant on AI to complete your academic assignments? | 1 2 3 4 5 |
| AID4 | Do you struggle to complete academic tasks without AI support? | 1 2 3 4 5 |
| AID5 | Do you feel the need to use AI more and more frequently to maintain your academic performance? | 1 2 3 4 5 |

**Personal Information**

Please mark the appropriate information for the following questions:

- Gender: Male; Female
- Student of the Year: 1. 1; 2. 2; 3. 3; 4. 4; 5. >=5
- Major: 1. Education; 2. Engineering; 3. Economic; 4. Language 5. Others
- Type of university: Private university; Public university
- Time to use AI: 1. Under 1 year; 2. 1 to Under 2 years 3. > 2 years

**THANK YOU SO MUCH!**
